# Ultrasonographic Findings in Fat Embolism Syndrome

**DOI:** 10.5811/cpcem.2021.2.51270

**Published:** 2021-03-12

**Authors:** Shun Yonezaki, Kazuya Nagasaki, Hiroyuki Kobayashi

**Affiliations:** Mito Kyodo General Hospital, Department of Internal Medicine, Ibaraki, Japan

**Keywords:** fat embolism syndrome, ultrasonographic findings

## Abstract

**Case Presentation:**

A 93-year-old man living in a nursing home presented to our emergency department with altered mental status. Examination revealed hypotension and severe hypoxia. Chest radiograph showed infiltrates in the right upper lobe, and computed tomography of the abdomen and pelvis demonstrated a left femoral neck fracture. A point-of-care transthoracic echocardiogram (TTE) revealed an enlarged right ventricle, severe tricuspid regurgitation, and numerous white floating dots moving toward the right atrium from the inferior vena cava (IVC), leading to the diagnosis of fat embolism syndrome (FES).

**Discussion:**

Although imaging studies can facilitate diagnosis, the diagnosis of FES is typically made by clinical history and presentation, making a swift diagnosis often difficult in those who are critically ill. Recent case reports have described that TTE can detect fat emboli, seen as flowing hyperechoic particles in IVC. This image demonstrates the utility of TTE to diagnose FES.

## CASE PRESENTATION

A 93-year-old man living in a nursing home presented to our emergency department with altered mental status, which occurred suddenly over the previous one hour. An initial history elicited from the nursing home staff failed to identify any risk factors. Examination revealed hypotension and severe hypoxia with oxygen saturation of 85% on 15 liters per minute oxygen with a non-rebreather mask. Chest radiograph showed infiltrates in the right upper lobe, but contrast computed tomography (CT) showed no pulmonary embolism. Suspecting an occult organ pathology, we ordered CT of the abdomen and pelvis, which demonstrated a left femoral neck fracture. Further inquiry of the nursing home staff revealed that the patient had fallen earlier on the same day, striking his hip on the floor. A point-of-care transthoracic echocardiogram (TTE) revealed an enlarged right ventricle, severe tricuspid regurgitation, and numerous white floating dots moving toward the right atrium from the inferior vena cava (IVC) (Image and Video), leading to the diagnosis of fat embolism syndrome (FES). Intravenous crystalloid and oxygen administration were initiated, but the patient expired five hours after admission despite supportive care.

## DISCUSSION

Fat embolism syndrome is a rare condition defined by the presence of fat globules in respiratory circulation.^1^ Fat embolism syndrome typically manifests after long bone and pelvic fractures. Hypoxemia, neurologic abnormalities, and petechial rash are the classic triad of FES^1^; however, none are specific for FES. There is no definitive treatment, although supportive care is the standard treatment.^2^ Although chest and brain imaging studies can facilitate diagnosis, the diagnosis of FES is typically made by clinical history and presentation, making a swift diagnosis often difficult in those who are critically ill. Previous reporting suggests that fat emboli can be detected using transesophageal echocardiography during orthopedic surgery.^3^ Recent case reports have described that TTE can detect fat emboli, seen as flowing hyperechoic particles in IVC.^4^ This image emphasizes the utility of TTE to diagnose FES.

**CPC-EM Capsule**

What do we already know about this clinical entity?

*Fat embolism syndrome (FES) is defined by the presence of fat globules in respiratory circulation; its diagnosis is often challenging.*

What is the major impact of the image(s)?

*Floating fat emboli can be detected using point-of-care ultrasound, which facilitates the diagnosis of FES.*

How might this improve emergency medicine practice?

*Point-of-care transthoracic ultrasound is an essential diagnostic tool to evaluate patients with suspected FES.*

## Supplementary Information

Video Transthoracic echocardiogram. The video shows numerous white floating dots moving toward the right atrium (RA) from the inferior vena cava (IVC) suggestive of fat embolism syndrome.

## Figures and Tables

**Image:**
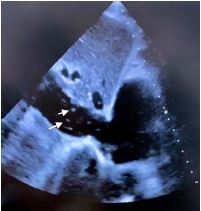
A transthoracic echocardiogram showing numerous white floating dots (arrows) in the inferior vena cava suggestive of fat embolism syndrome.
